# The Usefulness of Microcirculatory Assessment After Cardiac Surgery: Illustrative Case Report

**DOI:** 10.21470/1678-9741-2023-0383

**Published:** 2024-09-03

**Authors:** Marcos Fernandes, Andrea De Lorenzo, Eduardo Tibiriçá

**Affiliations:** 1 Instituto Nacional de Cardiologia, Ministry of Health, Rio de Janeiro, Rio de Janeiro, Brazil

**Keywords:** Microcirculation, MitralValve, Cardiac Surgical Procedures, Hemodynamics, Perfusion, Oxygen

## Abstract

Cardiac surgery causes a series of disturbances in human physiology. The correction of systemic hemodynamic variables is frequently ineffective in improving microcirculatory perfusion and delivering oxygen to the tissues. We present the case of a 52-year-old male submitted to mitral valve replacement (metallic valve) and subaortic membrane resection. Sublingual microcirculatory density and perfusion were evaluated using a handheld CytoCam camera before surgery and in the early postoperative period. In this case, systemic hemodynamic variables were compromised despite an actual improvement in the microcirculatory parameters in comparison to the preoperative evaluation, possibly due to the correction of the structural cardiac defects.

## INTRODUCTION

Cardiac surgical procedures may be lifesaving or significantly relieve symptoms and improve the quality of life of patients. There are several types of cardiac surgical procedures, and heart valve interventions due to rheumatic fever are more frequently performed in developing countries because the disease still has a high incidence in these regions^[1,2]^. Nonetheless, cardiac surgery causes a series of disturbances in human physiology as a result of several factors, including the need for cardiopulmonary bypass, which is essential for most invasive cardiac surgical procedures but causes a systemic inflammatory response in the peri- and postoperative periods^[3]^.

Hemodynamic and laboratory data are continuously monitored postoperatively in the intensive care unit (ICU). However, even though it is known that the maintenance of systemic microcirculation is critical for tissue metabolism, microcirculatory assessment is not routine. Ultimately, disturbances in the systemic microcirculation may result in inadequate blood supply to body tissues, which can lead to cell damage and multiple organ dysfunction. Importantly, parameters such as peripheral oxygen saturation and serum lactate are commonly used to assess tissue perfusion and provide only an indirect assessment of the microcirculation. Furthermore, it has been demonstrated that “hemodynamic incoherence” refers to the difference between the systemic microcirculatory flow and its global macrocirculatory counterpart^[1,2]^. Actually, the loss of hemodynamic coherence occurs when the correction of systemic hemodynamic variables is ineffective in improving microcirculatory perfusion and delivering oxygen to the tissues to preserve organ function^[1]^.

**Fig. 1:**
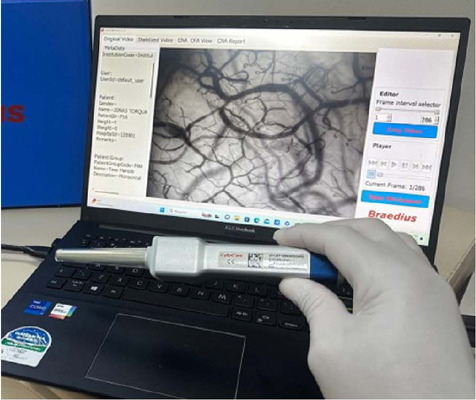
CytoCam-IDF handheld video microscope used for visualization of microcirculatory parameters in our department, based on IDF technology. The CytoCam is a pen-like device and is held as such. The low weight of the device (120 g) minimizes pressure artifact problems that were present in the earlier heavy devices. The camera is connected to a device controller based on a medical grade computer or a suitable portable device such as laptop or tablet, which is used for image storage (from https://braedius-medical.com/products/). IDF=incident dark field.

Therefore, we report the case of a cardiac postsurgical patient in whom the status of the microcirculation was assessed using real-time, noninvasive, point-of-care microcirculatory imaging of the sublingual microcirculation with an incident dark field camera (Figure 1) (Braedius Medical, Huizen, The Netherlands), as previously validated^[3,4,5]^. The sublingual region has been demonstrated to have a homogeneous spatial distribution for most microvascular parameters, including total and functional vascular density^[6]^.

## CASE PRESENTATION

A 52-year-old male who had previously undergone metallic aortic valve replacement due to rheumatic heart disease was admitted to a quaternary care, specialized cardiology hospital due to dyspnea (New York Heart Association class II) and recurrent syncope. Physical examination showed aortic and mitral murmurs, with clear lung fields. The electrocardiogram displayed sinus rhythm, with left atrial enlargement. Transthoracic echocardiogram showed biatrial enlargement, normal systolic left and right ventricular function, severe aortic stenosis and regurgitation, a subaortic membrane, and severe mitral stenosis and regurgitation.

The patient underwent mitral valve replacement (metallic valve) and subaortic membrane resection. The surgery was uneventful, the duration of extracorporeal circulation was 150 min, and the duration of aortic clamping was 139 min. He received six units of platelets, prothrombin complex, and fibrinogen. In the immediate postoperative period, complete atrioventricular block occurred, and an epicardial pacemaker was placed, followed by a permanent pacemaker.

**Fig. 2:**
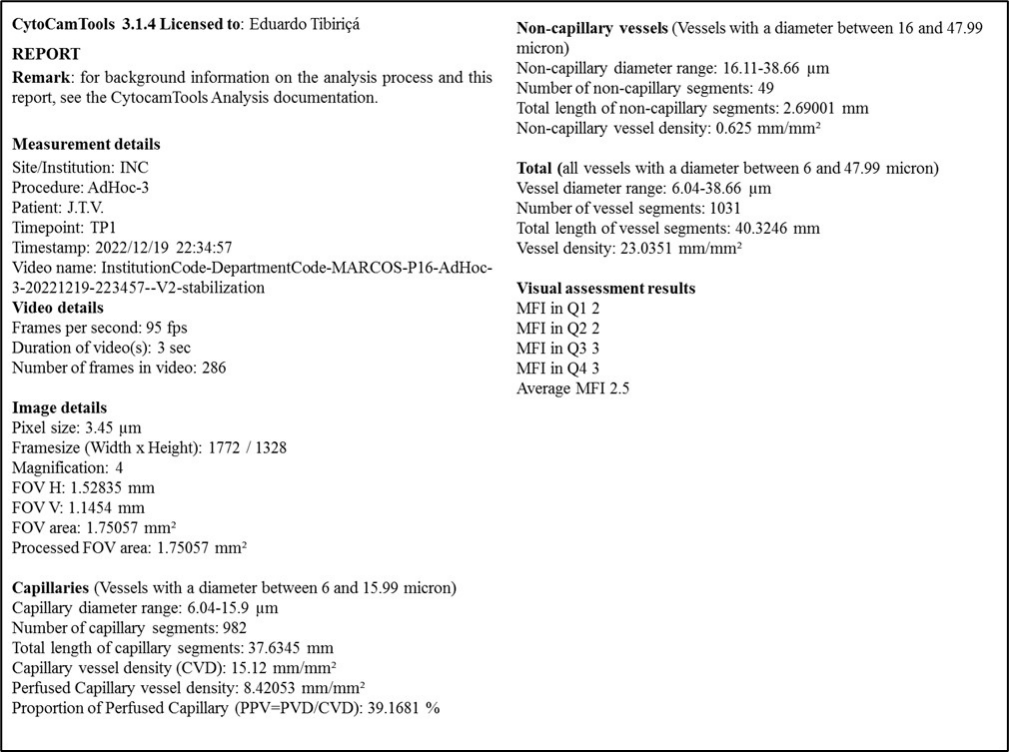
Representative example of the report of the offline image analysis performed using CytoCamTools 3.1.4 software (Braedius Medical, Huizen, The Netherlands).

**Fig. 3:**
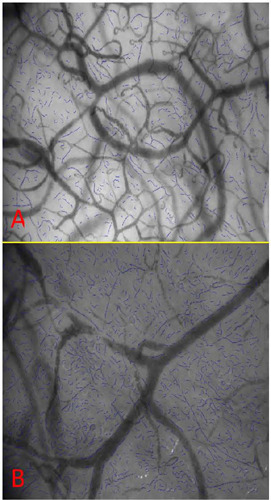
CytoCam-incident dark field imaging of the sublingual area of the patient using a handheld camera the day before heart valve surgery (A) and during the early postoperative period (B). The blue lines indicate capillary vessels (diameter range between 6 µm and 16 µm). Offline image analysis was performed using CytoCamTools 3.1.4 software (Braedius Medical, Huizen, The Netherlands). The number of capillary vessels increased after surgery, as shown in Figure 4.

**Fig. 4:**
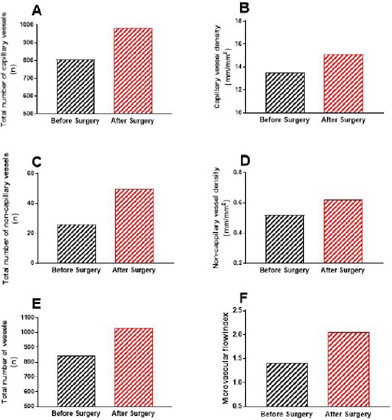
Bar graphs showing the analysis of the main microvascular parameters, including (A) total number of capillary vessels, (B) capillary vessel density, (C) total number of non-capillary vessels, (D) non-capillary vessel density, (E) total vessel number, and (F) microvascular flow index in the sublingual area, assessed using a handheld camera based on incident dark-field imaging before and after heart valve surgery.

The microcirculatory evaluation was performed twice — the day before cardiac surgery and then three hours after arrival to the ICU. At the preoperative evaluation, the patient reported that he had not taken any vasodilators or other medications with cardiovascular effects. His heart rate was 57 bpm, and his blood pressure was 117/72 mmHg. During the postoperative microcirculatory analysis, in the immediate postoperative period, the patient was sedated with dexmedetomidine (Precedex®, 0.2 µg/kg/h) and had a noradrenaline infusion (0.08 µg/kg/min). His heart rate was 79 bpm, and his blood pressure was 79/54 mmHg. At each microcirculatory evaluation, five videos (5-sec duration) were obtained, among which the best three (according to the microcirculation image quality score) were used for offline analysis using CytoCam Tools 3.1.4 software (Figure 2) (Braedius Medical, Huizen, The Netherlands). Figure 3 depicts the postoperative increase in capillary density that was observed in the patient. The microcirculatory analysis also included capillary vessels (diameter range between 6 µm and 16 µm) and non-capillary vessels (diameter range between 16 µm and 50 µm). The total number of vessels represents the total number of vessels with diameters < 50 µm. Figure 4 shows the main microcirculatory parameters obtained from the analysis. Additionally, the videos were analyzed in a blinded fashion for calculation of the microvascular flow index (Figure 4F), as previously described^[7]^. This is a semiquantitative score that distinguishes between no flow (0), intermittent flow (1), sluggish flow (2), and continuous flow (3). A score was assigned to each quadrant of the video screen. The scores of the four quadrants were averaged per video, and the values from three videos were averaged. Of note, all parameters were increased postoperatively.

## CONCLUSION

In this case, systemic blood pressure was relatively low post cardiac surgery, even with vasopressor support, thereby suggesting that the macrocirculation was compromised despite an actual improvement in the microcirculatory parameters in comparison to the preoperative evaluation, possibly due to the correction of the structural cardiac defects. This highlights the discrepancy, or incoherence, between macro and microcirculatory parameters, as previously described^[1]^. Indeed, as reported by De Backer et al.^[8]^, microcirculatory perfusion is usually maintained as long as the mean arterial pressure is over 65 mmHg^[9]^.

Traditional hemodynamic monitoring focuses on macrocirculatory parameters^[10]^. Nonetheless, the macrocirculatory profile may not reflect tissue perfusion, and even with adequate blood pressure and cardiac output, peripheral tissues may experience ischemia, leading to complications^[1]^. On the other hand, in patients with low blood pressure, reassurance about the status of the microcirculation may avoid unnecessary increases in vasopressor drug use, among other potentially harmful interventions. A better understanding of the microcirculatory status may therefore help to better manage such conditions and potentially improve outcomes.

## Abbreviations, Acronyms & Symbols

ICU: Intensive care unit
IDF: Incident dark field
IRB: Institutional Review Board
